# DZNep-mediated apoptosis in B-cell lymphoma is independent of the lymphoma type, EZH2 mutation status and MYC, BCL2 or BCL6 translocations

**DOI:** 10.1371/journal.pone.0220681

**Published:** 2019-08-16

**Authors:** Chidimma Agatha Akpa, Karsten Kleo, Dido Lenze, Elisabeth Oker, Lora Dimitrova, Michael Hummel

**Affiliations:** 1 Department of Experimental Hematopathology, Institute of Pathology, Charité Medical University, Berlin, Berlin, Germany; 2 Berlin School of Integrative Oncology, Charité Medical University, Berlin, Berlin, Germany; European Institute of Oncology, ITALY

## Abstract

Enhancer of zeste homolog 2 (EZH2) tri-methylates histone 3 at position lysine 27 (H3K27me3). Overexpression and gain-of-function mutations in EZH2 are regarded as oncogenic drivers in lymphoma and other malignancies due to the silencing of tumor suppressors and differentiation genes. EZH2 inhibition is sought to represent a good strategy for tumor therapy. In this study, we treated Burkitt lymphoma (BL) and diffuse large B-cell lymphoma (DLBCL) cell lines with 3-deazaneplanocin—A (DZNep), an indirect EZH2 inhibitor which possesses anticancer properties both in-vitro and in-vivo. We aimed to address the impact of the lymphoma type, EZH2 mutation status, as well as MYC, BCL2 and BCL6 translocations on the sensitivity of the lymphoma cell lines to DZNep-mediated apoptosis. We show that DZNep inhibits proliferation and induces apoptosis of these cell lines independent of the type of lymphoma, the EZH2 mutation status and the MYC, BCL2 and BCL6 rearrangement status. Furthermore, DZNep induced a much stronger apoptosis in majority of these cell lines at a lower concentration, and within a shorter period when compared with EPZ-6438, a direct EZH2 inhibitor currently in phase II clinical trials. Apoptosis induction by DZNep was both concentration-dependent and time-dependent, and was associated with the inhibition of EZH2 and subsequent downregulation of H3K27me3 in DZNep-sensitive cell lines. Although EZH2, MYC, BCL2 and BCL6 are important prognostic biomarkers for lymphomas, our study shows that they poorly influence the sensitivity of lymphoma cell lines to DZNep-mediated apoptosis.

## Introduction

EZH2 is a histone modifier that plays an important part in tumor initiation, development, progression, metastasis, and drug resistance [1]. EZH2 is the core component of polycomb repressive complex 2 (PRC2) responsible for its histone lysine methyltransferase catalytic activity [2–4]. It is known that EZH2 is overexpressed in a variety of malignancies including some types of lymphomas, and gain-of-function mutations involving Tyr646 (previously Tyr641), Ala682 (previously Ala677) and Ala692 (previously Ala687) have been reported for this gene, resulting in increased tri-methylation of H3K27 [5–10]. The increased tri-methylation of H3K27 created by enhanced EZH2 activity, results in repression of tumor suppressor and differentiation genes, which can drive tumor formation, progression and metastasis [11–13]. Hence, inhibiting EZH2 can be a successful strategy for treatment of lymphoma with EZH2 alterations. Several direct EZH2 inhibitors have been developed and their efficacy for the induction of apoptosis in lymphoma cell lines was demonstrated, however, most of these direct inhibitors induce apoptosis preferably in cell lines bearing EZH2 point mutations [14, 15]. 3-Deazaneplanocin A (DZNep) is an indirect EZH2 inhibitor, which not only prevents tri-methylation of H3K27, but also inhibits migration and proliferation, as well as induces cell death in many cancer cell lines and primary tumor cells [16–23]. Moreover, the H3K27me3 demethylation exerted by DZNep causes the reactivation of a set of PRC2-repressed genes in cancer cells, thus, effecting apoptosis whilst sparing normal cells [16]. Hence, the potential for clinical usage of DZNep has been discussed [24, 25]. DZNep indirectly inhibits EZH2 by blocking the enzyme S-adenosylhomocysteine hydrolase (AHCY) which plays an important role in the DNA methylation process. The inhibition of AHCY by DZNep causes impediment of S*-*adenosyl-methionine (AdoMet)—dependent methyl transferase reactions due to the intracellular build-up of S-adenosylhomocysteine (AdoHcy) levels [24]. This in turn lifts the transcriptional repression of tumor suppressor genes posed by the H3K27me3 mark in the genome. We sought to investigate the correlation between the type of aggressive B-cell lymphoma, the EZH2 mutation status, and the relationship between MYC, BCL2 and BCL6 translocations–the latter with high impact on the prognosis and for guiding decisions for treatment [26–28]—and the sensitivity of lymphoma cell lines to apoptosis upon DZNep treatment.

## Materials and methods

### Cell lines and cell culture

Seven diffuse large B-cell lymphoma (DLBCL) cell lines HT (ACC-567), WSU-DLCL-2 (ACC-575), SU-DHL-10 (ACC-576), CARNAVAL (ACC-724), U-2932 R1, U-2932 R2, KARPAS-422 (ACC-32), and five Burkitt lymphoma (BL) cell lines BLUE-1 (ACC-594), CA-46 (ACC-73), DG-75 (ACC-83), BL-2 (ACC-625), BL-41 (ACC-160) were used. All cell lines were obtained from Deutsche Sammlung von Mikroorganismen und Zellkulturen GmbH (DSMZ), Braunschweig, Germany. U-2932 R1 and U-2932 R2 sub clones were donated by Dr. Hilmar Quentmeier [29], Leibniz-institute DSMZ, Braunschweig, Germany. They were grown in RPMI 1640 medium (Life Technologies GmbH/ Thermo Fisher Scientific, Darmstadt, Germany) supplemented with 20% fetal calf serum (FCS) (PAN-Biotech, Aidenbach, Germany). All cell lines were incubated at 37°C and 5% CO_2_.

### Drug preparations

DZNep (Selleckchem, Munich, Germany) was dissolved in sterile water, and EPZ-6438 (Selleckchem, Munich, Germany) was dissolved in dimethyl sulfoxide (DMSO) (SERVA Electrophoresis GmbH, Heidelberg, Germany) as recommended by the manufacturer. Both drugs were diluted with the respective solvents and stored at -20°C until used for the treatment of cell lines.

### Apoptosis measurement

For measurement of apoptotic cells, flow cytometry using the BD Accuri C6 flow cytometer (BD Biosciences, California, USA) was performed. Briefly, 3 x 10^5^ cells from the well-mixed culture were washed twice with cell staining buffer (Biolegend, BIOZOL Diagnostica Vertrieb GmbH, Eching, Germany). The supernatant was discarded and the cells stained with a mixture of annexin V (Ann V) binding buffer (BD Biosciences, California, USA), propidium iodide (PI) (Biolegend, California, USA) and allophycocyanin (APC) annexin V (Biolegend, California, USA) at a ratio of 20:2:1 respectively. After a 10-minute incubation step at room temperature, the number of apoptotic cells was calculated using the BD Accuri C6 software (BD Biosciences, California, USA). The fraction of cells gated to indicate apoptosis include the Ann V/PI-positive cells.

### Cell proliferation assay

The respective cell lines were seeded at an initial cell density of 2 x 10^5^ cells / ml in cell culture flasks and treated with 5 μM DZNep, or without DZNep. The number of vital cells were counted every 24 hours, 48 hours and 72 hours using flow cytometry with appropriate gating to exclude apoptotic and dead cells.

### Inhibitory concentration 50 (IC_50_) determination

The IC_50_ was defined as the concentration of DZNep required to decrease cell viability by 50%. Cell lines were treated in 6-well cell culture plates with increasing concentrations of DZNep ranging from 0.5 μM until 10 μM. The percentage of annexin V/PI positive (non-viable) cells was measured afterwards. A plot of the percentage of non-viable cells versus concentration of DZNep was made and the IC_50_ was calculated for each cell line using linear regression.

### Western blotting

3 x 10^6^ cells were harvested and washed with 1x Gibco Dulbecco’s phosphate buffered saline (DPBS) (Life Technologies GmbH/ Thermo Fisher Scientific, Darmstadt, Germany). Whole cell lysis was done with 200 μl RIPA buffer (Merck KGaA, Darmstadt, Germany) and 1x Complete protease inhibitor cocktail (Roche Diagnostics GmbH, Mannheim, Germany). These were added to the cells and the mixture incubated for 30 minutes on ice. Afterwards, 2 cycles of high power sonication with Bioruptor Sonicator (Diagenode, Liège, Belgium) was applied onto the cells. A centrifugation step for 15 minutes at 13,000 revolutions per minute was performed and the supernatant was collected and used for protein concentration determination. The rest of the lysate was stored at -80°C prior to use. The protein concentration was measured using the Pierce Bicinchoninic acid (BCA) protein assay Kit (Thermo Fisher Scientific, Darmstadt, Germany), following the manufacturers protocol. 10–15 μg of the protein lysate was separated on a 12% Expedeon RunBlue SDS protein gel (Biozol Diagnostica Vertrieb GmbH, Eching, Germany). The protein was transferred onto a 0.2 μM Immobilon-P-membrane (Serva Electrophoresis, Heidelberg, Germany) by electroblotting. After transfer, the membrane was blocked in 5% milk solution (dissolved in PBST) for 1 hour. The membranes were incubated with the primary antibodies overnight at 4°C. After washing the membrane 3 times for 5 minutes in PBST, the secondary antibody was applied. Incubation of the membrane with the secondary antibody was done at room temperature for 1 hour. Afterwards, the membranes were once again washed in PBST three times for 5 minutes. Immobilion Forte Western HRP substrate (Millipore corporation, Massachusetts, USA) was added onto the blot to aid chemiluminiscence. Signals were detected using the FUSION-SL-3500 system (Peqlab Biotechnologie, Erlangen, Germany). Image analysis and signal quantification was done using the FUSION-CAP Software (Vilber Lourmat, Eberhardzell, Germany). Primary antibodies used for Western blot include anti-glyceraldehyde 3-phosphate dehydrogenase (GAPDH) antibody (catalogue number: 2118S), anti-EZH2 antibody (catalogue number: 5246S), anti- H3K27me3 antibody (catalogue number: 9733S), and anti-cleaved PARP antibody (catalogue number: 9541S) (all obtained from Cell Signaling Technology, Frankfurt am Main, Germany). The secondary antibody used was anti-rabbit HRP-conjugated (catalogue number: NA934V) (GE Healthcare, Illinois, USA).

### DNA isolation, RNA extraction and cDNA synthesis

Genomic DNA was extracted from cells using QIAamp DNA Mini Kit, and RNA was isolated from cells using the RNeasy Midi Kit (both from Qiagen, Hilden, Germany) following the recommendations of the manufacturer. For cDNA synthesis, total RNA was treated with DNase I (Thermofisher Scientific, Darmstadt, Germany) for 30 minutes at 37°C. To the DNA-digested product, 50 mM EDTA was added and incubated for 10 minutes at 65°C. Total RNA was reverse transcribed with a T3 thermocycler (Biometra GmbH, Jena, Germany) to cDNA using TaqMan Reverse Transcription reagents and random hexamer primers (ThermoFisher Scientific, Darmstadt, Germany). Cycling conditions used include primer annealing at 25°C for 10 minutes, DNA polymerization at 37°C for 30 minutes and finally reverse transcriptase deactivation at 95°C for 5 minutes. The synthesized cDNA was stored at -20°C pending further use.

### Primer design, PCR and Sanger sequencing

EZH2 primers were designed using the NCBI Primer BLAST software (National Center for Biotechnology Information, U.S. National Library of Medicine, Bethesda MD, USA). The forward and reverse primer sequence for the EZH2 genomic region of exon 16 include *5’-TCCCCAGTCCATTTTCACC-3’* and *5’-TCATTTCCAATCAAACCCACAG-3’* respectively (product length = 256 base pairs). For detection of EZH2 point mutations at the RNA (cDNA) level, the forward and reverse primer sequence utilized for Sanger sequencing include 5’-*TGACCTCTGTCTTACTTGTGGAG-3’* and 5’-*GCAGTTTGGATTTACCGAATG-3’* respectively. This primer sequence covers the EZH2 mutation hotspots on exon 16 and 18, with a product length of 340 base pairs. PCR was performed on the ProFlex PCR system Thermocycler (Applied Biosystems / Thermo Fisher Scientific, Darmstadt, Germany). 10 μl of the respective PCR products was mixed with 2 μl 6x Gel loading dye (New England Biolabs Inc., Massachusetts, USA) and loaded onto 7% polyacrylamide gels. 6 μl Gene Ruler low range DNA ladder (Thermo Fisher Scientific, Darmstadt, Germany) was also loaded onto the gel. For the run, 1x Tris-borate-EDTA (TBE) buffer was used, and gels were set to run for 30 minutes at 150 Volts. The gel image was developed upon staining with ethidium bromide (Sigma-Aldrich Biochemie GmbH, Hamburg, Germany), and the image was captured using the Gel Doc 2000 (Bio-Rad laboratories GmbH, Berlin, Germany). The PCR products were cleaned using the Wizard SV Gel and PCR Clean-Up System (Promega Corporation, Madison, USA) following the manufacturers instruction. The concentration of the cleaned-up PCR product was measured using the NanoDrop ND-1000 (Kisker-Biotech, Steinfurt, Germany). Sanger sequencing was done by LGC Genomics GmbH, Berlin, Germany.

### Fluorescence in situ hybridization (FISH) analysis

FISH was performed on formalin-fixed paraffin-embedded (FFPE) tissue sections and cytospins of cell lines as already described [30, 31]. Probes used include Vysis LSI dual color break-apart probes for MYC (catalogue number: 01N63-020) and BCL6 (catalogue number: 01N23-020) (both purchased from Vysis/Abbott Molecular Diagnostics, Wiesbaden-Delkenheim, Germany), as well as BCL2 FISH DNA Probe, Split signal (catalogue number: Y5407) (purchased from Dako/Agilent, Hamburg, Germany). Analysis WSU-DLCL-2 and HT for MYC translocation was not performed as this has already been reported [32, 33]. The FISH probes were hybridized onto their respective target chromosomes using DakoCytomation Hybridizer, (Dako/Agilent, Hamburg, Germany). Visualization and analysis was done using the Zeiss Axio Imager Z1 (Zeiss, Jena, Germany) and the Isis imaging software version 5.3.1 (Metasystems, Altlussheim, Germany) respectively.

### Statistical analysis

Statistical analysis was done using the GraphPad Prism 5 software (GraphPad Software, California, USA). Statistical significance between groups was determined using the Mann-Whitney U test (two-tailed) and p values < 0.05 were considered significant.

## Results and discussion

The functional significance of EZH2 overexpression and gain-of-function mutations in cancer has been extensively studied [34–40]. Its resultant effect made manifest by alterations in the expression of genes regulating apoptosis, cell proliferation, migration and differentiation [1, 41]. Inhibition of EZH2 is thus important for the epigenetic regulation of lymphoma progression mediated by EZH2, given that these gain-of-function mutations occur in lymphomas with an incidence of up to 7% - 25% [5, 9]. In line with published data [5, 42], we also show from the group of cell lines we analyzed, that the EZH2 Tyr646 mutation could occur more frequently in lymphoma when compared with the Ala682 and Ala692 point mutations (Table 1).

**Table 1:** Correlation between the lymphoma type, EZH2 mutation status, MYC, BCL2 and BCL6 rearrangements and DZNep sensitivity.

Cell line	Lymphoma type	EZH2 Tyr646 mutation	Type of Tyr646 mutation	MYC rearrangement	BCL2 rearrangement	BCL6 rearrangement	DZNep sensitive	IC_50_
**Carnaval**	**DLBCL**	**+**	**TAC→TTC (Tyr641Phe)**	**+**	**+**	**-**	**+**	**2.5**
**U-2932 R2**	**DLBCL**	**-**	**-**	**+**	**-**	**-**	**+**	**3.0**
**BL-41**	**BL**	**-**	**-**	**+**	**-**	**-**	**+**	**3.8**
**BL-2**	**BL**	**-**	**-**	**+**	**-**	**-**	**+**	**3.8**
**SU-DHL-10**	**DLBCL**	**+**	**TAC→TTC (Tyr646Phe)**	**+**	**+**	**-**	**+**	**5.4**
**KARPAS-422**	**DLBCL**	**+**	**TAC→AAC (Tyr646Asn)**	**-**	**+**	**-**	**+**	**6.3**
**BLUE-1**	**BL**	**-**	**-**	**+**	**-**	**-**	**+**	**8.7**
**WSU-DLCL-2**	**DLBCL**	**+**	**TAC→TTC (Tyr646Phe)**	**+**	**+**	**+**	**+**	**10.1**
**U-2932 R1**	**DLBCL**	**-**	**-**	**-**	**-**	**-**	**+**	**13.2**
**HT**	**DLBCL**	**-**	**-**	**-**	**-**	**-**	**-**	**17.8**
**DG-75**	**BL**	**-**	**-**	**+**	**-**	**-**	**-**	**27.4**
**CA-46**	**BL**	**-**	**-**	**+**	**-**	**-**	**-**	**33.4**

Sanger sequencing was performed using genomic DNA and RNA (cDNA) isolated from 12 B-cell lymphoma cell lines. This analysis was confined to 3 frequent mutation hot spots on exon 16 (Tyr646) and 18 (Ala682 and Ala692). Mutations on Ala682 and Ala692 were not detected in any of the cell lines analyzed.

**Fig 1:**
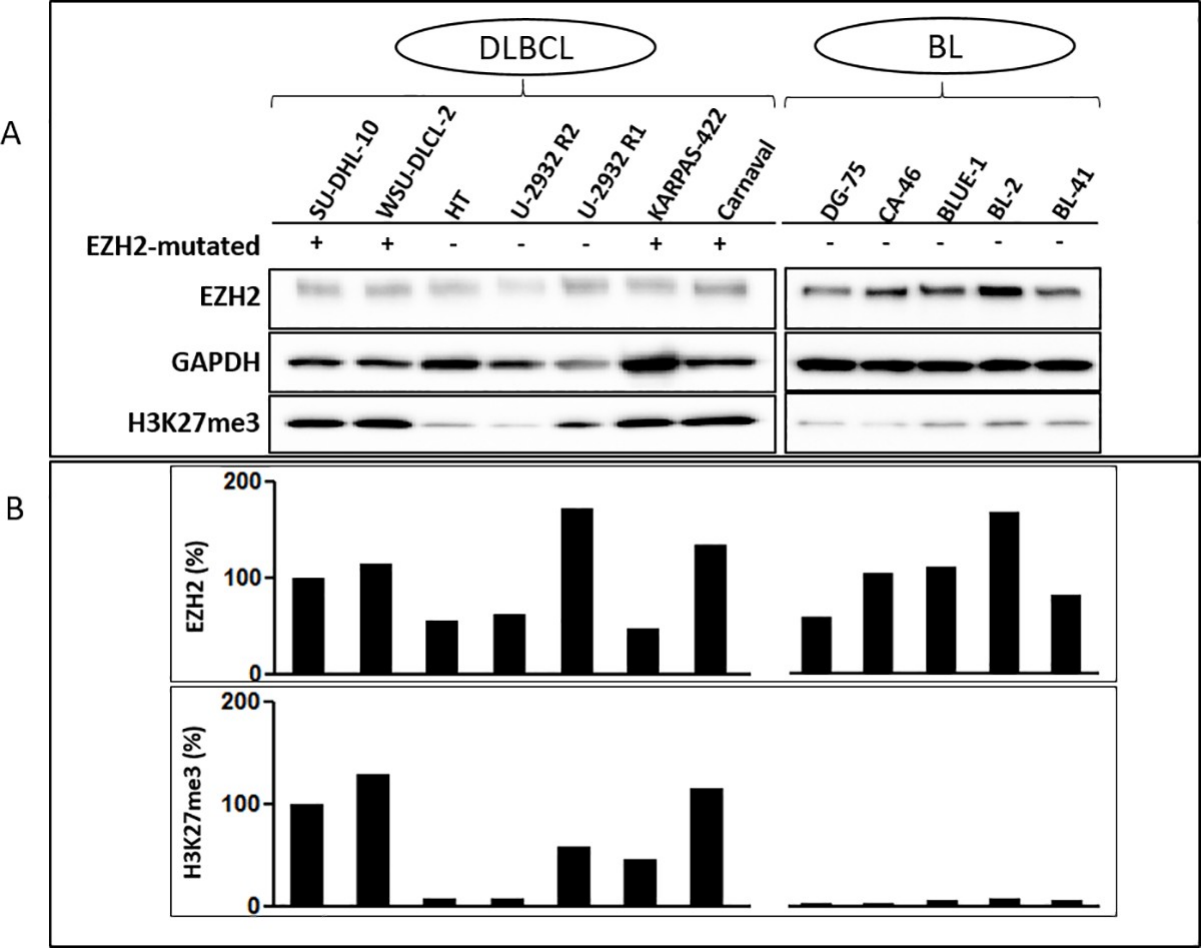
Effect of mutated EZH2 on H3K27me3 protein expression in B-cell lymphoma. (A) Western blot performed using total protein lysates from EZH2-mutated (SU-DHL-10, WSU-DLCL-2, KARPAS-422, Carnaval) and wild-type EZH2 (HT, U-2932 R2, U-2932 R1, DG-75, CA-46, BLUE-1, BL-2 and BL-41) cell lines showing an elevated H3K27me3 in EZH2-mutated cell lines as compared to wild-type EZH2 cell lines. (B) Densitometric quantification of EZH2 and H3K27me3 normalized for GAPDH.

This heterozygous Tyr646 point mutation has also been demonstrated by other reports investigating the role of these gain-of-function mutations in primary tumor patient samples [5, 9, 42–45]. From our data, this mutation is present, and it is expressed in DLBCLs, particularly those with BCL2 translocations in agreement with previous studies [9, 43, 46]. This gain-of-function mutation causes increased H3K27me3 (Fig 1). The consequence of which has been published to promote transcriptional repression [45–49].

To establish optimal conditions for growth of the cell lines with and without DZNep pressure, and to show when the cell lines begin to exhibit an apoptotic phenotype, we made concentration- and time-course experiments. We sought a situation where one could optimally measure apoptosis upon DZNep treatment, while excluding external apoptosis-inducing factors such as overcrowding of the cells in the culture flask.

**Fig 2:**
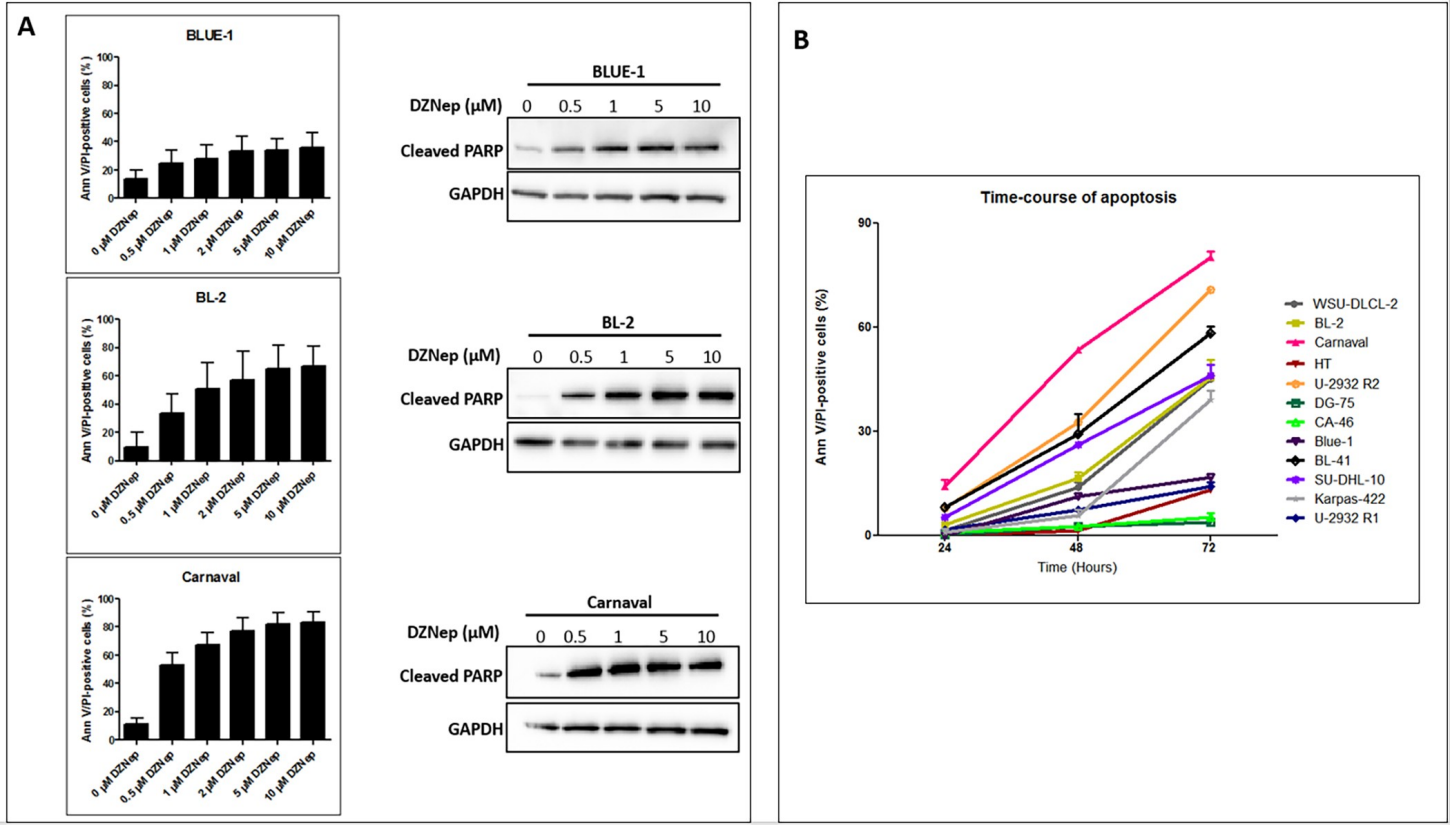
Apoptosis mediated by DZNep is concentration-dependent and time-dependent. (A) Flow cytometry and Western blot analysis showing apoptosis of 3 susceptible cell lines treated with increasing concentrations of DZNep for 72 hours. Flow cytometry data were obtained from 3 biological replicates and are shown as mean plus standard deviation (SD). (B) Lymphoma cell lines were treated with 5 μM DZNep and apoptosis was measured at 24 hours, 48 hours and 72 hours. The values shown are those normalized with that of the respective controls, and represent the mean plus standard deviation from triplicate measurements.

We reveal that cell death caused by DZNep is concentration-dependent (Fig 2A), increasing progressively with the DZNep concentration up to 5 μM. There was no marked difference in the percentage of apoptotic cells using 10 μM DZNep. Apoptosis was shown using flow cytometry employing annexin V/PI staining and Western blotting for detection of cleaved PARP. We also observe that at about 72 hours post exposure to DZNep, there was marked apoptosis in majority of the cell lines, with a high percentage of apoptotic cells measured for the DZNep-sensitive cell lines (Fig 2B).

Prolonged treatment of cell lines past 72 hours was not recommended because of overgrowth of the controls past the recommended maximum cell density provided by the DSMZ. Our results corroborate similar concentration and time-dependence of apoptosis mediated by DZNep in colon cancer cell lines [50] and non-small cell lung cancer cell lines [51].

**Fig 3:**
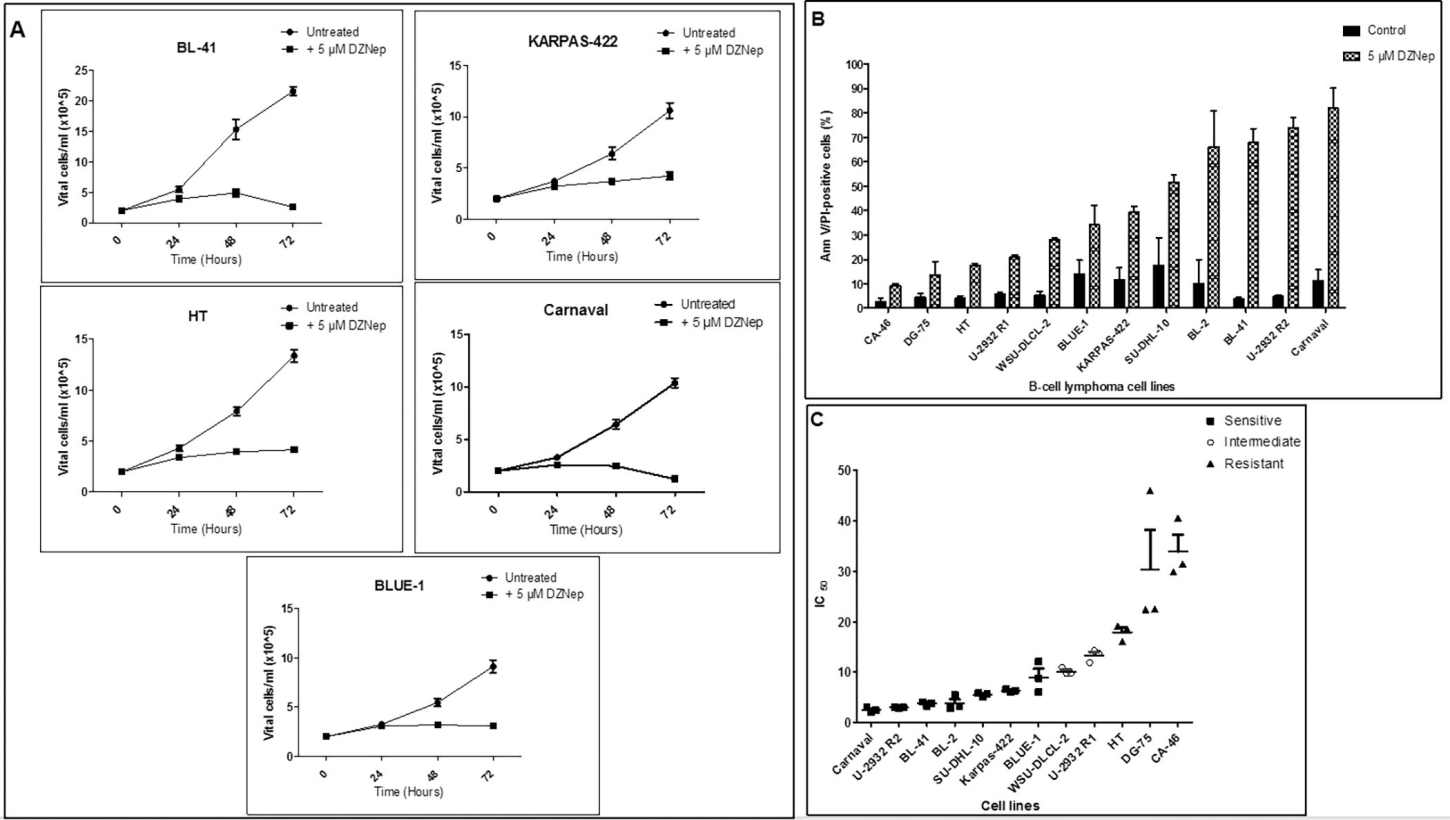
Impact of DZNep treatment on proliferation and apoptosis. (A) Cell lines were seeded at a density of 2 x 10^5^ vital cells / ml. They were either untreated or treated with 5 μM DZNep on day 0. The number of vital cells was determined after 24 hours, 48 hours and 72 hours. The number of vital cells was recorded after exclusion of the apoptotic / dead cell population (annexin V/PI positive cells) by flow cytometry. (B) Cell lines were either untreated or treated with 5 μM DZNep for a duration of 72 hours. The percentage of apoptotic cells was measured using flow cytometry. (C) IC_50_ determination and grouping of the cell lines into DZNep-sensitive, an intermediate and DZNep-resistant. For Fig 3A, 3B and 3C, data is shown as mean plus SD, n = 3 biological replicates.

Moreover, we show that the indirect EZH2 inhibitor, DZNep, inhibits proliferation (Fig 3A) and induces apoptosis (Fig 3B) in aggressive B-cell lymphoma cell lines, in conformation with similar reports on other cancer cell lines [22, 23, 51, 52]. This is also true for primary tumor cells [17], and in-vivo in mice [18]. It is also obvious that DZNep impairs proliferation in some lymphoma cell lines for example; BLUE-1 and KARPAS-422 whereas, apoptosis in these cell lines only seem to be moderately affected. Nevertheless, although majority of the cell lines were sensitive to apoptosis mediated by DZNep, not all the lymphoma cell lines underwent marked apoptosis. This is not unexpected because, we are aware of the issue of drug resistance during cancer therapy [53, 54], occurring via various mechanisms. To better classify the cell lines used in this study as DZNep-sensitive or DZNep-resistant, we performed an IC_50_ experiment. The IC_50_ is the concentration of DZNep that decreases the percentage of viable cells of a given cell line by 50%. From our data (Fig 3C), we uncovered 3 groups of cell lines; 7 DZNep-sensitive cell lines with a calculated IC_50_ within the range of 2–10 μM, 2 intermediate-responding cell lines with IC_50_ values between 10–15 μM and 3 resistant cell lines with IC_50_ values above 15 μM DZNep.

**Fig 4:**
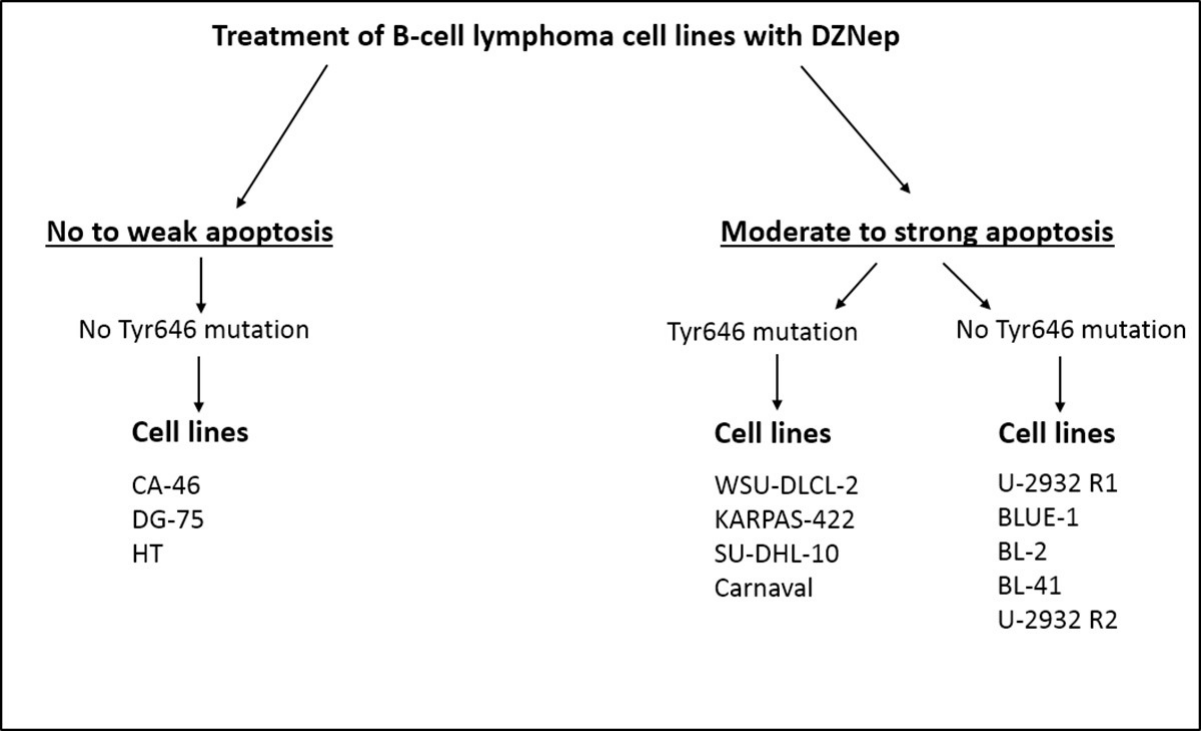
Grouping of wild-type and EZH2 mutated lymphoma cell lines based on their response to apoptosis caused by DZNep.

Furthermore, we establish that the sensitivity of lymphoma cell lines to DZNep-induced apoptosis does not depend on the EZH2 mutation status of the cell lines (Fig 4), as it does with other EZH2 inhibitors, for example, CPI-360 [55], EPZ-6438 [56] and GSK126 [57]. Nonetheless, we notice that all DZNep-resistant cell lines lacked this mutation. This implies that DZNep has no tendency towards selectively causing apoptosis in lymphoma cell lines with mutated EZH2.

We also investigated the relationship between DZNep sensitivity of the cell lines, and the lymphoma type, the MYC (8q24), BCL2 (18q21) and BCL6 (3q27) translocations. For the latter purpose, we performed FISH analysis with the respective fluorescence-labeled probes on the cell lines. A summary of our findings is shown in Table 1.

Our results reveal no particular trend associating the DZNep sensitivity or resistance of the cell lines with the type of lymphoma, or translocations involving the genes for MYC, BLC2 or BCL6. This shows that the lymphoma type and these biomarkers are poor indicators of the effectiveness of DZNep in inducing apoptosis in B-cell lymphomas.

**Fig 5:**
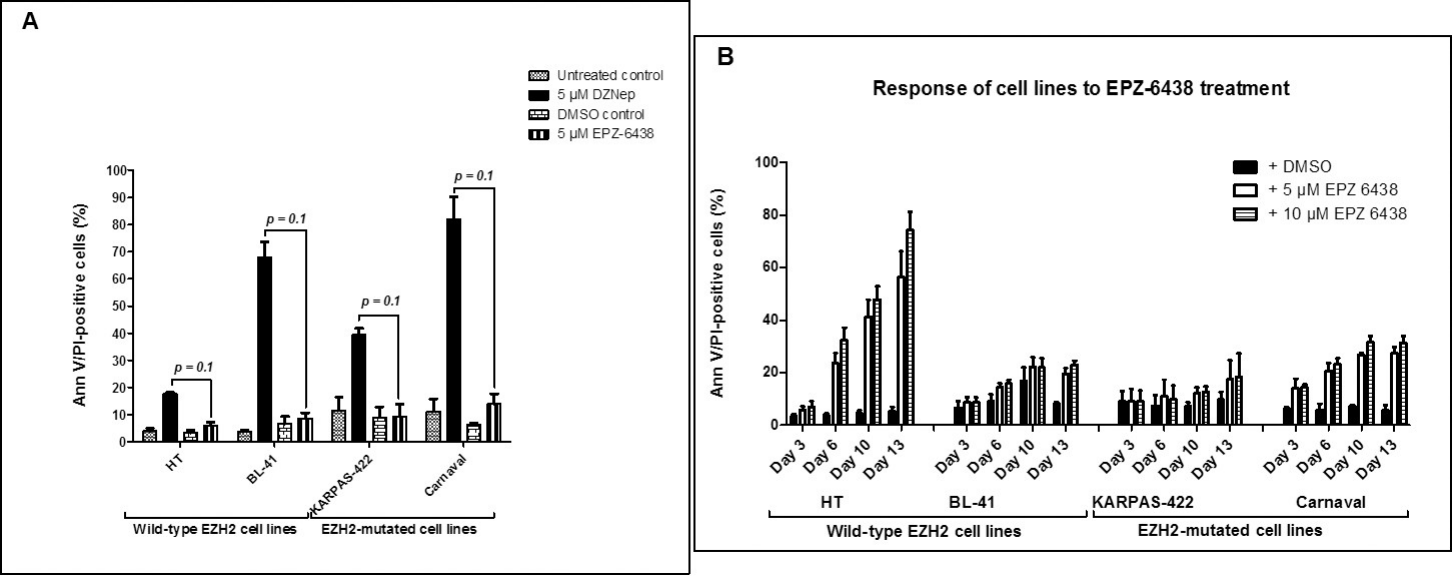
DZNep induces stronger apoptosis at a lower concentration and within a shorter period than EPZ-6438. (A) One DZNep-resistant wild-type EZH2 cell line (HT), two DZNep-sensitive EZH2-mutated cell lines (Carnaval and KARPAS-422) and one DZNep-sensitive wild-type EZH2 cell line (BL-41) were treated with 5 μM DZNep and 5 μM EPZ-6438 for 3 days. Apoptosis was measured afterwards by flow cytometry. Data was analyzed using GraphPad Prism 5 software (GraphPad Software, California, USA) and statistical significance was determined using the Mann-Whitney U test (n = 3 biological replicates). (B) The same cell lines as were treated with 5 μM and 10 μM EPZ-6438 for up to 13 days. Apoptosis measurement, splitting the cells, and medium change were done on day 3, day 6, day 10 and day 13. Fig 5A and 5B are shown as mean plus SD from three biological replicates.

We compared the apoptotic effect of DZNep and a direct EZH2 inhibitor EPZ-6438 (also known as Tazemetostat) which is currently in phase II clinical trials for patients with solid tumors **(***ClinicalTrials*.*gov identifier (NCT number)*: **NCT02601950)**. First, we treated 2 EZH2- mutated (Carnaval and KARPAS-422) and 2 EZH2 wild-type (HT and BL-41) cell lines with 5 μM DZNep and 5 μM EPZ-6438 for 72 hours, and determined the percentage of apoptotic cells. We showed that DZNep causes apoptosis in both wild-type and EZH2-mutated cell lines, showing no preference for each case. In addition, DZNep caused obvious apoptosis (p = 0.1) of DZNep-sensitive cell lines under similar conditions in comparison with EPZ-6438 (Fig 5A). We also noticed a change of about 10% in the percentage of apoptotic cells measured upon treatment of the DZNep-resistant cell line HT with DZNep and EPZ-6438 for 72 hours respectively. To check how these cell lines respond to apoptosis upon extended EPZ-6438 pressure, we treated the same cell lines as those latter mentioned with 5 μM and 10 μM EPZ-6438. To be consistent with a previous study [56], the cell lines were treated with EPZ-6438 for up to a duration of 13 days. Apoptosis measurement, splitting of the cells and changing of culture medium was done every 3–4 days. Following each medium change, the equivalent amount of EPZ-6438 was replaced in the culture flask. We observe a comparable percentage of apoptosis for the DZNep-sensitive, EZH2-mutated and wild-type cell lines (Fig 5B). For the DZNep-resistant, wild-type EZH2 cell line (HT) however, we see a pronounced increase in apoptosis, starting from day 6 and reaching almost 80% at day 13. This is in contrast to some previous studies in which EPZ-6438 has been described to selectively kill lymphoma cells bearing the EZH2 mutation, with minimal effects on wild-type EZH2 lymphoma cells [56, 58]. Our data suggests that both lymphoma cell lines with mutated and wild-type EZH2 are sensitive to EPZ-6438. This is in line with the interim report from the on-going phase II multi-center study where EPZ-6438 treatment showed an overall response rate of 71% in lymphoma patients with mutated EZH2 and 33% in patients with wild-type EZH2 [59].

**Fig 6:**
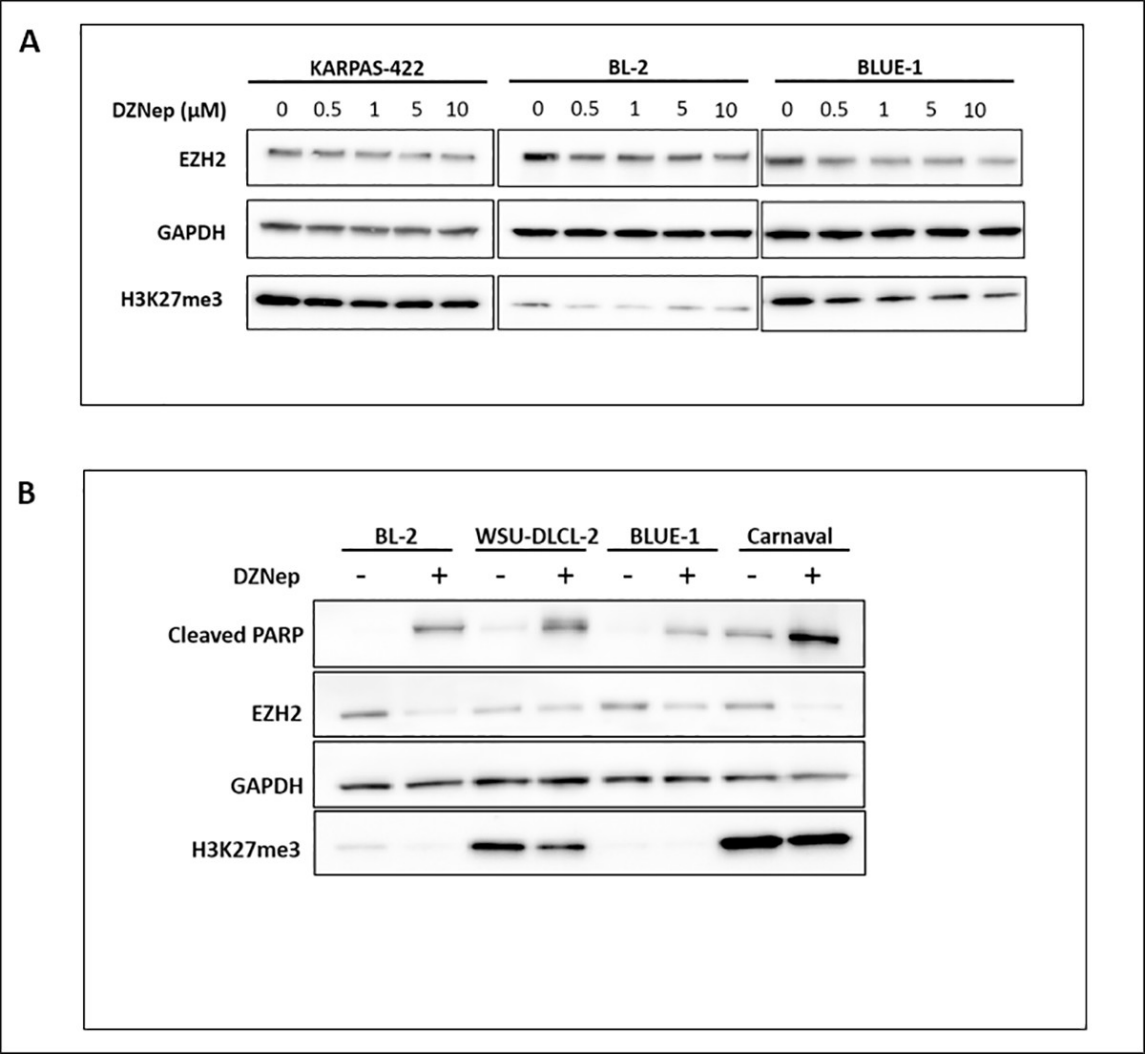
Apoptosis caused by DZNep occurs via EZH2 inhibition and downregulation of H3K27me3. (A) Downregulation of EZH2 and H3K27me3 in three DZNep-sensitive lymphoma cell lines treated with increasing concentration of DZNep. Western blot was performed using total protein lysates from the cell lines. (B) Western blot showing the treatment of two EZH2-mutated (WSU-DLCL-2 and Carnaval) and two wild-type EZH2 (BL-2 and BLUE-1) cell lines with DZNep.

As with cell lines of other cancer entities such as chondrosarcoma [23], breast and colon cancer [16], DZNep inhibits EZH2 and promotes the downregulation of H3K27me3 in the three DZNep-sensitive lymphoma cell lines treated with DZNep (Fig 6A). This indicates that both EZH2 and H3K27me3 are biological targets of DZNep. In addition, we demonstrate that although cell lines with EZH2 gain-of-function mutation have increased H3K27me3, DZNep still lowers this tri-methylation by inhibiting EZH2 and promotes apoptosis in these cells (Fig 6B).

Since some cell lines remained resistant to DZNep despite its strong apoptotic capacity, further investigation into the molecular mechanism of resistance to DZNep is recommended, as this would enable the identification of biomarkers that could be of therapeutic relevance for EZH2 inhibition by DZNep.

## Conclusions

Our work reveals the anti-tumor properties of DZNep and shows its strong apoptotic effects in aggressive B-cell lymphoma cell lines. We furthermore provide evidence that the increased EZH2 function mediated by mutated EZH2 does not decrease the ability of DZNep to inhibit H3K27me3 and induce apoptosis in the cell lines. In addition, although MYC, BCL2 and BCL6 rearrangements are important prognostic markers for patients with DLBCL, the sensitivity of lymphoma cell lines to DZNep-mediated apoptosis is independent of these rearrangements. Finally, the type of lymphoma, BL or DLBCL is not predictive for the success of DZNep treatment. We are currently working on the identification of alternative biomarkers.

## Supporting information

S1 Fig Sanger sequencing data of the four lymphoma cell lines (SU-DHL-10, KARPAS-422, Carnaval and WSU-DLCL-2) with expressed EZH2 Tyr646 mutation. (PDF)

S2 Fig FISH data from lymphoma cell lines showing the presence (+) or absence (-) of (A) MYC break, (B) BCL2 rearrangement and (C) BCL6 translocations. Presence of MYC translocation in SU-DHL-10, DG-75 and CA-46 is documented in the DSMZ data sheet for the cell lines (see DSMZ nos.: ACC 576, ACC 83 and ACC 73 respectively).
(PDF)
