# Supplementary material for: DZNep-mediated apoptosis in B-cell lymphoma is independent of the lymphoma type, EZH2 mutation status and MYC, BCL2 or BCL6 translocations
Source: PLoS One. 2019 Aug 16;14(8):e0220681. doi: 10.1371/journal.pone.0220681 (PMC6697340; doi:10.1371/journal.pone.0220681)
Supplement: S1 Fig — (PDF) [file pone.0220681.s001.pdf]

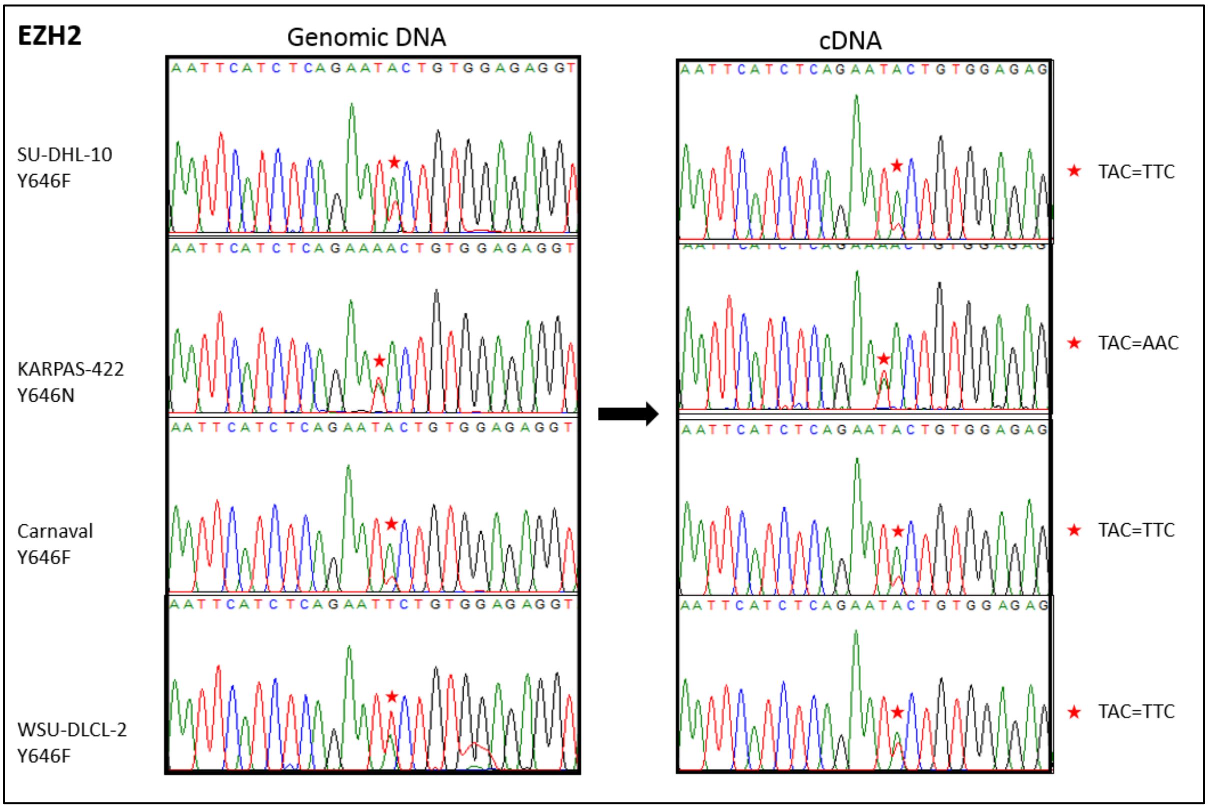

S1 Fig. Sanger sequencing data of the four lymphoma cell lines (SU-DHL-10, KARPAS-422, Carnaval and WSU-DLCL-2) with expressed EZH2 Tyr646 mutation.
