# Supplementary material for: DZNep-mediated apoptosis in B-cell lymphoma is independent of the lymphoma type, EZH2 mutation status and MYC, BCL2 or BCL6 translocations
Source: PLoS One. 2019 Aug 16;14(8):e0220681. doi: 10.1371/journal.pone.0220681 (PMC6697340; doi:10.1371/journal.pone.0220681)
Supplement: S2 Fig — FISH data from lymphoma cell lines showing the presence (+) or absence (-) of (A) MYC break, (B) BCL2 rearrangement and (C) BCL6 translocations. Presence of MYC translocation in SU-DHL-10, DG-75 and CA-46 is documented in the DSMZ data sheet for the cell lines (see DSMZ nos.: ACC 576, ACC 83 and ACC 73 respectively). (PDF) [file pone.0220681.s002.pdf]

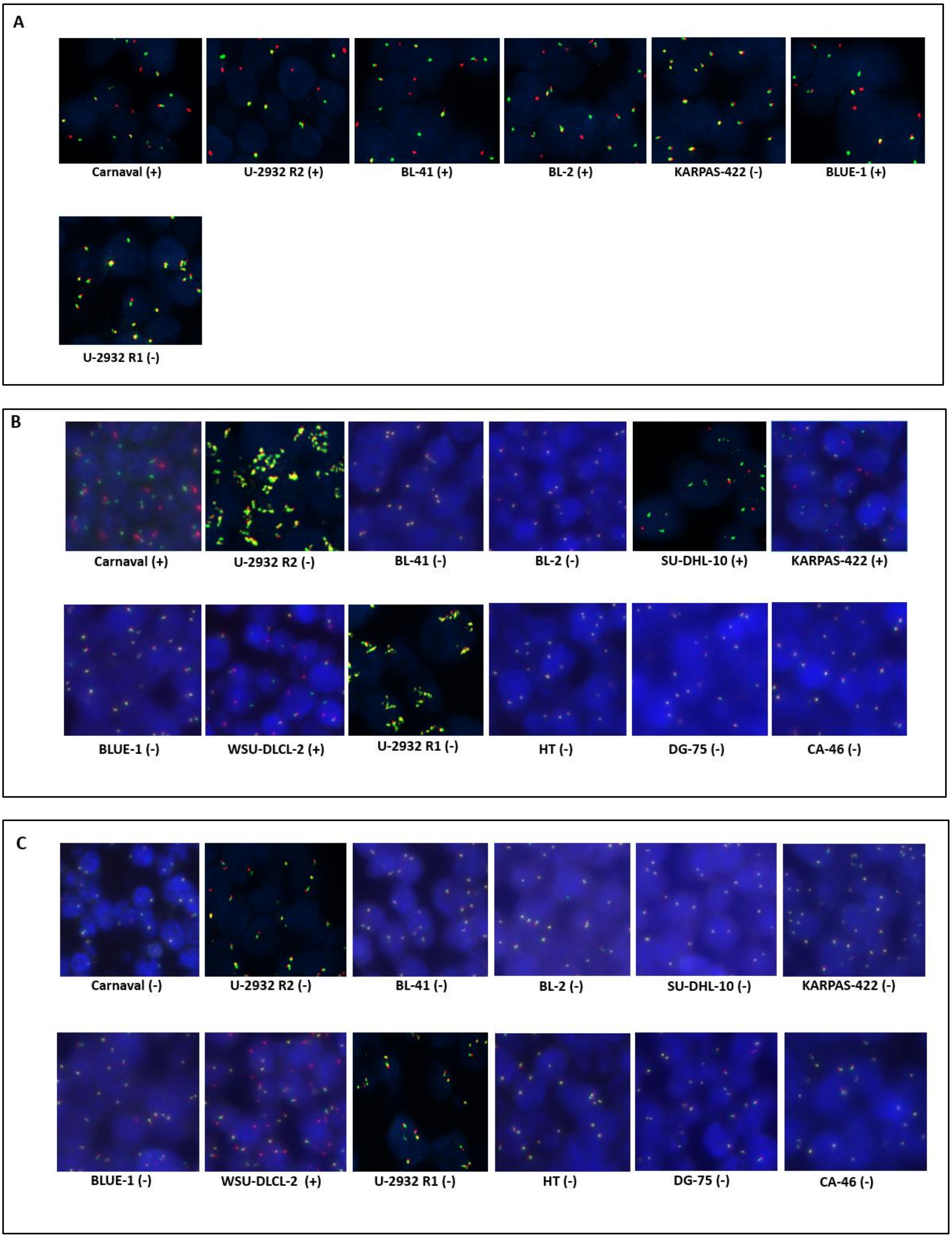

S2 Fig. FISH data from lymphoma cell lines showing the presence (+) or absence (-) of (A) MYC break, (B) BCL2 rearrangement and (C) BCL6 translocations. Presence of MYC translocation in SU-DHL-10, DG-75 and CA-46 is documented in the DSMZ data sheet for the cell lines (see DSMZ nos.: ACC 576, ACC 83 and ACC 73 respectively).
